# Editorial: Harnessing the gut microbiome: paving the way for personalized treatment of diverse disorders

**DOI:** 10.3389/fmed.2026.1946088

**Published:** 2026-09-16

**Authors:** Devi Lal, Aneta Sokal-Dembowska, Sara Jarmakiewicz-Czaja

**Affiliations:** 1 Department of Zoology, Ramjas College, University of Delhi, Delhi, India; 2 Faculty of Health Sciences and Psychology, Collegium Medicum, University of Rzeszów, Rzeszów, Poland; 3 University Center for Research and Development in Health Sciences, University of Rzeszów, Rzeszów, Poland

**Keywords:** gut microbiome, gut microbiota modulation, personalized dietary interventions, personalized treatment, precision medicine

The gut microbiome is a complex ecosystem of trillions of microorganisms that maintains intestinal barrier homeostasis and, consequently, good health (1). Precision medicine is gaining importance in the context of analyzing “omic” data from the gut microbiome, including information on the composition of the gut microbiota in both health and disease, its metabolites, and functional genes (2). The research topic “*Harnessing the Gut Microbiome: Paving the Way for Personalized Treatment of Diverse Disorders*” aimed to explore how insights into the gut microbiome can inform personalized medicine approaches for various disorders. The research topic included three original research articles, one review article, one case report, and one perspective.

Modeling the composition of the gut microbiota may offer potential for personalized dietary interventions, which in turn may lead to increased treatment efficacy for certain diseases. A given microbiome intervention does not work the same way for every patient, and outcomes improve when treatment accounts for a patient's specific microbial, metabolic, or immune profile. Jia et al. showed this directly in patients with ulcerative colitis (UC), where combining probiotics and a low-FODMAP (fermentable oligosaccharides, disaccharides, monosaccharides, and polyols) diet led to greater improvements in clinical symptoms and a reduction in inflammatory markers in patients with mild-to-moderate UC. However, the authors pointed out that the use of a low-FODMAP diet alone may potentially reduce the number of beneficial gut bacteria, therefore, the best results are observed when it is used in conjunction with probiotic therapy.

The review from Luo et al. on gut microbiota modulation in irritable bowel syndrome (IBS) made the same point in a different setting. Given the various subtypes of IBS, therapy selection, whether probiotics or fecal microbiota transplantation (FMT), needs to be matched precisely to the individual patient.

Precision microbiome treatment depends on having reliable biomarkers. Sun et al. examined bacterial, fungal, and viral signatures in chronic functional constipation and found that bacterial and viral signatures allow for the differentiation of CFC, whereas the fungal signature provided limited diagnostic information. The study's findings indicate that a multi-kingdom approach is crucial for identifying new microbiome-based biomarkers.

Xiong et al. added a related finding where a correlation between gut microbiota patterns at 21 days of life in preterm infants and the development of bronchopulmonary dysplasia (BPD) was examined. They found that infants enriched with pathobionts showed a higher rate of BPD. Since the sample size in this study was very small, larger studies are needed to confirm this relationship.

The final two papers examined how far microbiome-targeted therapy can be extended to non-intestinal disease. Despite the numerous benefits of modulating the gut microbiome, careful patient selection and monitoring of FMT safety are essential, which once again emphasizes the need for individualized treatment. In a case report, Peng et al. reported that the use of FMT in children with autism spectrum disorder (ASD) resulted in anaphylactic shock, suggesting that children with ASD and multiple food intolerances carry this risk.

In a perspective article, Sun and Yang indicated that gut dysbiosis and damage to the intestinal barrier increase antigen translocation and immune system activation and that Gd-IgA1 forms immune complexes with antibodies that deposit in the kidneys, leading to damage to the glomeruli. The authors propose precise targeting of the gut-kidne*y* axis, including modulating the composition of the gut microbiota, supporting the restoration of the intestinal barrier, and providing precise immunomodulation, which would constitute promising therapeutic strategies.

There is an agreement that microbiome-based interventions need to be individualized, but there are certain gaps that remain. Several of the studies were small or exploratory, which limits how confidently their findings can be applied to clinical decisions. Further research is needed so that the diagnostic panels can separate informative signals, such as the bacterial and viral markers in constipation, from less useful ones, such as the fungal signature in that same study. Findings from small or single-center studies, particularly those linking early-life microbiota composition to later disease, need validation in larger, multi-site cohorts before clinical application. Biomarker discovery would also benefit from treating multi-omic profiling, bacterial, fungal, and viral data together. As FMT and related interventions reach new patient groups, including children with atopic or allergic conditions, pre-treatment risk screening needs to catch up with where the therapy is heading. Getting these things right would help turn the clinical insights in this Research Topic into treatments that are both effective and matched to the right patients.

The use of gut microbiome modulation opens up new possibilities for the development of personalized therapeutic and dietary strategies for various diseases, including several without an obvious gut origin. Getting there will take bigger validation studies, biomarker panels that look beyond bacteria alone, and closer attention to which patients are likely to benefit or be harmed by a given intervention.
